# Ultrasonic Sensor Signals and Optimum Path Forest Classifier for the Microstructural Characterization of Thermally-Aged Inconel 625 Alloy

**DOI:** 10.3390/s150612474

**Published:** 2015-05-27

**Authors:** Victor Hugo C. de Albuquerque, Cleisson V. Barbosa, Cleiton C. Silva, Elineudo P. Moura, Pedro P. Rebouças Filho, João P. Papa, João Manuel R. S. Tavares

**Affiliations:** 1 Programa de Pós-Graduação em Informática Aplicada, Universidade de Fortaleza, Fortaleza, Ceará 60811-905, Brazil; E-Mail: cleissonvb@gmail.com; 2 Departamento de Engenharia Metalúrgica e de Materiais, Universidade Federal do Ceará, Fortaleza, Ceará 60455-900, Brazil; E-Mails: cleiton@ufc.br (C.C.S.); elineudo@ufc.br (E.P.M.); 3 Programa de Pós-Graduação em Ciências da Computação, Instituto Federal de Educação, Ciência e Tecnologia do Ceará, Fortaleza, Ceará 61939-140, Brazil; E-Mail: pedrosarf@ifce.edu.br; 4 Departamento de Ciência da Computação, Universidade Estadual Paulista, Bauru, São Paulo 17033-360, Brazil; E-Mail: papa@fc.unesp.br; 5 Instituto de Ciência e Inovação em Engenharia Mecânica e Engenharia Industrial, Departamento de Engenharia Mecânica, Faculdade de Engenharia, Universidade do Porto, Porto 4200-465, Portugal; E-Mail: tavares@fe.up.pt

**Keywords:** ultrasonic sensor, metric function, optimum path forest, signal classification, microstructural characterization

## Abstract

Secondary phases, such as laves and carbides, are formed during the final solidification stages of nickel-based superalloy coatings deposited during the gas tungsten arc welding cold wire process. However, when aged at high temperatures, other phases can precipitate in the microstructure, like the *γ”* and δ phases. This work presents an evaluation of the powerful optimum path forest (OPF) classifier configured with six distance functions to classify background echo and backscattered ultrasonic signals from samples of the inconel 625 superalloy thermally aged at 650 and 950 °C for 10, 100 and 200 h. The background echo and backscattered ultrasonic signals were acquired using transducers with frequencies of 4 and 5 MHz. The potentiality of ultrasonic sensor signals combined with the OPF to characterize the microstructures of an inconel 625 thermally aged and in the as-welded condition were confirmed by the results. The experimental results revealed that the OPF classifier is sufficiently fast (classification total time of 0.316 ms) and accurate (accuracy of 88.75% and harmonic mean of 89.52) for the application proposed.

## 1. Introduction

Nb-bearing nickel-based superalloys, in particular inconel 625, has greater applicability, especially in highly corrosive environments, such as the ones in the oil and gas industry, than many other nickel (Ni)-based alloys. Nowadays, this alloy is used widely in the weld overlay of the inner surface of carbon steel pipes and other equipment of the offshore industry. However, further studies about this alloy, such as the one presented in this paper, are necessary to increase the overall knowledge of its properties.

During the welding of an inconel 625 alloy, there is an intensive microsegregation of some elements, such as niobium (Nb) and molybdenum (Mo), within the interdendritic regions, causing the supersaturation of the liquid metal with these chemical elements in its final stage of solidification, which results in the precipitation of the Nb-rich laves phase and MCprimary carbides of type NbC [1,2]. This segregation and precipitation of the secondary phases can change the mechanical properties of the alloy and decrease its resistance to corrosion [3]. In addition, the Nb-rich laves phase has a low melting point that causes an increase in the temperature solidification range, making the alloy susceptible to solidification cracking [4].

Nondestructive testing based on ultrasonic signals has been commonly used to study this kind of material. For example, in the evaluation of the embrittlement kinetics and elastic constants of the SAF2205 duplex stainless steel for different aging times at 425 and 475 °C [5], spinodal decomposition mechanism study on the UNSS31803 duplex stainless steel [6], evaluation of grain refiners' influence on the mechanical properties in a CuAlBe shape memory alloy [7], sigma phase detection on a UNS S31803 duplex stainless steel [8], characterization of welding defects [9], characterization of cast iron microstructure [10], pattern classification in nondestructive materials inspection [11], nondestructive characterization of microstructures and determination of elastic properties in plain carbon steel [12] and in the phase transformations evaluation on a UNS S31803 duplex stainless steel based on nondestructive testing [13].

In this sense, the main goal of this work was to evaluate the influence of six distance functions, mainly the Euclidean, chi-square, Manhattan, Canberra, squared chi-squared and Bray–Curtis distances, in the performance of the recent and powerful optimum path forest classifier to detect/identify, based on ultrasonic signals, the kinetics of the phase transformation of a Ni-based alloy thermally aged at 650 and 950 °C for 10, 100 and 200 h, as well as in the as-welded state. Raw data ultrasonic background echo and backscattered signals acquired with two types of transducers (4 and 5 MHz) were used. For a further assessment of the distance functions' performance, the results obtained were very satisfactory in terms of accuracy rate, train and test times, confusion matrix and harmonic mean between specificity and sensitivity, which makes the results presented and discussed of noteworthy value.

The OPF has been evaluated in different applications as, for example, EEG signal classification for epilepsy diagnosis [14], ECG arrhythmia classification [15], automatic characterization of graphite particles in metallographic images [16], intrusion detection in computer networks [17], aquatic weed automatic classification [18] and spoken emotion recognition [19], among others.

## 2. Materials and Methods

This section describes the experimental work done for the temperatures of 650 and 950 °C for 10, 100 and 200 h, as well as for the as-welded state. First, the ultrasonic sensor signals acquired and the related fundamentals are introduced. Afterwards, the optimum path forest classifier used to classify the ultrasonic signals is presented. Finally, the metrics used in the classifier evaluation are described.

### 2.1. Ultrasonic Sensor Signals

After the welding and preparation of the samples, described in detail in [20,21], the background echo and backscattered signals were acquired to evaluate the effect of aging on the inconel 625 alloy samples.

The pulse echo technique and the direct contact method were used to collect the background echo and backscattered ultrasonic signals [8]. All of the signals were obtained using commercial nondestructive testing (NDT) ultrasonic transducers: one of 4 MHz (Krautkramer, Model MB4S, Lewistown, PA, USA) and another one of 5 MHz (Krautkramer, Germain, Model MSW-QCG). The choice of these transducers was based on the authors previous experience in this kind of NDT and knowledge concerning the material under study [22–25]. In fact, Albuquerque *et al.*, in [21], showed that these frequencies revealed were to be the most adequate to analyze the material under study, as a transducer with a frequency of 10 MHz completely attenuated the ultrasonic signal; and one with a frequency of 2.25 MHz led to an adjacent echo that overlapped the signal extensively, seriously compromising the accuracy of the results.

As a coupling material, the SAE 15W40 lube oil was used for the longitudinal measurements. A Krautkramer ultrasonic device (GE Inspection Technologies, Lewistown, PA, USA, model USD15B) was used connected to a 100-MHz digital oscilloscope (Tektronix, Portland, OR, USA, model TDS3012B), which transmitted the ultrasonic signals to a computer for processing, according to a sampling rate of 1 GS/s.

The microstructural characterization was carried out using the OPF classifier configured with the Euclidean, chi-square, Manhattan, Canberra, squared chi-squared and Bray–Curtis distances on the original background echo and backscattered signals. In order to assure statistical significance, 40 signals were acquired for each sample, and each background echo signal had 10,000 points; *i.e.*, a total of 400,000 points was attained, and each backscattered signal had 500 points, resulting in a total of 20,000 points for this study.

Albuquerque *et al.*, in [21], did not consider echo signals without preprocessing, claiming that the large number of points made their use impracticable. However, this problem has been overcome, because the classifier used here is faster and more powerful, which is one of the important contributions attained with this work. Nunes *et al.* [20] compared the OPF, configured only with the Euclidean distance, with the support vector machine and Bayesian classifiers and showed its superiority in terms of the processing time and accuracy rate. Thus, another contribution of this work was to analyze the influence of six distance functions on the OPF's performance to detect/identify microstructural changes from the ultrasonic signals due to aging.

### 2.2. Optimum Path Forest Classifier

The OPF classifier models the problem of pattern recognition as a graph partition in a given feature space. The nodes are represented by the ultrasonic signal feature vectors, and all pairs are connect by edges, defining a complete graph. This kind of representation is straightforward, given that the graph does not need to be explicitly represented, and has low memory requirements. The partition of the graph is carried out by a competition process between some key samples, known as prototypes, which offer optimum paths to the remaining nodes of the graph. Each prototype sample defines its optimum path tree (OPT), and the collection of all OPTs defines the optimum path forest, which gives the name to the classifier [26].

The OPF can be seen as a generalization of the well-known Dijkstra algorithm to compute optimum paths from a source node to the remaining ones [27]. The main difference relies on the fact that OPF uses a set of source nodes, *i.e.*, the prototypes, with any path-cost function. In the case of Dijkstra's algorithm, a function that summed the arc-weights along a path was applied. For OPF, a function that gives the maximum arc-weight along a path is used [26].

Let *Z* = *Z*_1_ ∪ *Z*_2_ be a dataset labeled with a function λ, in which *Z*_1_ and *Z*_2_ are, respectively, training and test sets, and let *S* ⊆ *Z*_1_ be a set of prototype patterns (ultrasonic signal feature vectors). Essentially, the OPF classifier builds a discrete optimal partition of the feature space, such that any sample *s* ∈ *Z*_2_ can be classified according to this partition. This partition is an optimum path forest (OPF) computed in ℜ*^n^* by the image foresting transform (IFT) algorithm [28].

The OPF algorithm may be used with any smooth path-cost function that can group ultrasonic signal features with similar properties [28]. This work used the path-cost function *f_max_*, which is computed as:

(1) $$\begin{array}{ll} f_{\max}\left(\langle s\rangle \right) & =\{\begin{array}{ll} 0 & \text{if}s\in S,\\ +\infty & \text{otherwise}. \end{array}\\ f_{\max}\left(\pi \cdot \langle s,t\rangle \right) & =\max\left\{f_{\max}\left(\pi \right),d\left(s,t\right)\right\} \end{array}$$

in which *d*(*s, t*) means the distance between ultrasonic signal features *s* and *t*, and a path *π* is defined as a sequence of adjacent features. As such, *f_max_*(*π*) computes the maximum distance between adjacent samples in *π*, when *π* is not a trivial path.

The OPF algorithm assigns one optimum path P*(*s*) from *S* to every ultrasonic signal feature *s* ∈ *Z*_1_, originating an optimum path forest *P* (a function with no cycles, which assigns to each *s* ∈ *Z*_1_\*S* its predecessor *P*(*s*) in P*(*s*) or a marker *nil* when *s* ∈ *S*. Let *R*(*s*) ∈ *S* be the root of P*(*s*) that can be reached from *P*(*s*). The OPF algorithm computes for each *s* ∈ *Z*_1_ the cost *C*(*s*) of P*(*s*), the label *L*(*s*) = λ(*R*(*s*)) and the predecessor *P*(*s*).

The OPF classifier is composed of two distinct phases: (I) training; and (II) classification. The former step consists, essentially, of finding the prototypes and computing the optimum path forest, which is the union of all OPTs rooted at each prototype. After that, a sample is picked from the test sample, which connects it to all of the samples of the optimum path forest generated in the training phase. Notice that this test sample is not permanently added to the training set, *i.e.*, it is performed only once. The next sections describe this procedure in more detail.

### 2.3. Training

We say that S* is an optimum set of prototypes when the OPF algorithm minimizes the classification errors for every *s* ∈ *Z*_1_. S* can be found by exploiting the theoretical relation between the minimum spanning tree (MST) and optimum path tree for *f_max_* [29]. The training essentially consists of finding S* and an OPF classifier rooted at S*.

By computing an MST in the complete graph (*Z*_1_, *A*), we obtain a connected acyclic graph whose nodes are all ultrasonic signal features of *Z*_1_, and the arcs are undirected and weighted by the distances *d* between adjacent features. The optimum spanning tree is the tree that has the least sum of its arc compared to any other spanning tree in the complete graph. In the MST, every pair of ultrasonic signal features is connected by a single path that is optimum according to *f_max_*. That is, the minimum spanning tree contains one optimum path tree for any selected root node.

The optimum prototypes are the closest elements of the MST with different labels in *Z*_1_; *i.e.*, elements that fall in the frontier of the classes. By removing the arcs between different classes, their adjacent features become prototypes in S*, and OPF can compute an optimum path forest with minimum classification errors in *Z*_1_. It should be noted that a given class may be represented by multiple prototypes, *i.e.*, optimum path trees, and there must exist at least one prototype per class.

### 2.4. Classification

For any ultrasonic signal feature *t* ∈ *Z*_2_, all arcs connecting *t* with samples *s* ∈ *Z*_1_ are addressed, as though *t* were part of the training graph. Considering all possible paths from S* to *t*, the optimum path P*(*t*) from S* is found, and *t* is labeled with the class λ(*R*(*t*)) of its most strongly connected prototype *R*(*t*) ∈ S*. This path can be identified incrementally by evaluating the optimum cost *C*(*t*) as:

(2) $$\begin{array}{cc} C\left(t\right)=\min\left\{\max\left\{C\left(s\right),d\left(s,t\right)\right\}\right\}, & \forall s \end{array}\in Z_{1}$$

Let the node s* ∈ *Z*_1_ be the one that satisfies Equation (2), *i.e.*, the predecessor *P*(*t*) in the optimum path P*(*t*). Given that *L*(s*) = λ(*R*(*t*)), the classification simply assigns *L*(s*) as the class of t. An error occurs when *L*(s*) = λ(*t*).

### 2.5. Performance Evaluation Metrics

In order to analyze the performance of the machine learning technique used, three metrics were employed: accuracy, sensitivity and specificity.

Accuracy (*Acc*) is defined as the ratio of the total number of samples correctly classified and the number of total samples,

(3) $$\text{Accuracy}=\frac{\text{number}\;of\;\text{correctly}\;\text{classified}\;\text{samples}}{\text{number}\;\text{total}\;\mathrm{of}\;\text{samples}}$$

Sensitivity (*Se*) can be defined as the ratio of the total number of samples correctly classified of one class and the total number of samples classified as belong to that class, including the number of the missed classified samples,

(4) $$\text{Sensitivity}=\frac{\text{true}\;\text{positives}}{\text{true}\;\text{positives}+\text{false}\;\text{negatives}}$$

in which true positives and false negatives stand for the number of samples of a given class correctly and incorrectly classified, respectively.

Specificity (*Sp*) stands for the ratio of the total number of samples correctly classified and the number of all samples classified as belonging to a specific class,

(5) $$\text{Specificity}=\frac{\text{true}\;\text{negatives}}{\text{true}\;\text{negatives}+\text{false}\;\text{positives}}$$

in which true negatives stands for the number of samples not belonging to a given class classified as not belonging to the considered class, while false positives stands for the number of samples incorrectly classified as belonging to a given class. Observe that these last two measures are based on the data of each class.

Furthermore, we also propose the use of a harmonic average between sensitivity and specificity, that is the harmonic mean (*HM*):

(6) $$HM=2\times \frac{Se\times Sp}{Se+Sp}$$

These evaluation metrics can be computed from a confusion matrix, which can be obtained by comparing the expected classification (reference data) with the ones predicted by the classifier. Besides these measures for evaluating and comparing the effectiveness performance of the classifier used, we also compute the training and testing times.

## 3. Results and Discussion

The original ultrasonic background echo and backscattered signals, acquired using 4- and 5-MHz transducers, were classified using the OPF classifier configured with the Euclidean, chi-square, Manhattan, Canberra, squared chi-squared and Bray–Curtis distances. The classification efficiency (processing time) and efficacy (accuracy rate, confusion matrices and harmonic mean) were analyzed. Thus, it is possible to evaluate the performance of the classifier to identify the microstructural classes. The original signals, for all distance metrics, were partitioned using the holdout method (50% for training and 50% for testing). The standard deviation for the mean accuracy, harmonic mean and processing time load over 10 iterations generated randomly were computed. The execution was performed on a personal computer with an Intel Core i3, at 3 GHz and with 3 GB of RAM using Linux Ubuntu as the operational system.

### 3.1. Efficiency and Effectiveness Analysis

The performance of each distance used by the OPF classifier was assessed through the accuracy rate, harmonic mean between specificity and sensitivity, processing time considering the training and testing phases and, finally, the confusion matrix.

**Figure 1:**
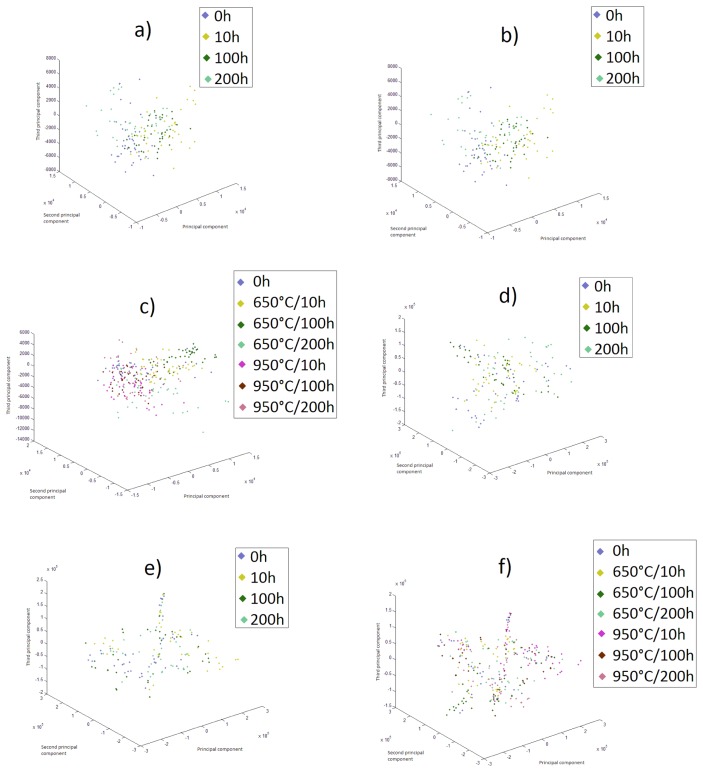
Spatial distribution of the samples of the (**a**) 4-MHz backscattered signals at 650 °C, (**b**) 950 °C and (**c**) of both temperatures together and of the (**d**) pulse echo signals at 650 °C, (**e**) 950 °C and (**f**) of both temperatures together.

Figure 1 shows the PCA (principal component analysis) corresponding to the spatial distribution of the samples of the 4-MHz backscattered signals at 650 °C (Figure 1a), 950 °C (Figure 1b) and both temperatures together (Figure 1c), as well as for the background pulse echo signals for 650 °C (Figure 1d), 950 °C (Figure 1e) and both temperatures together (Figure 1f). Accordingly, it is possible to analyze the complexity during the difficult assignment of classifying the samples.

#### 3.1.1. Aged Samples at 650 °C

In Table 1, which shows the accuracy rate and harmonic mean for the 4- and 5-MHz frequency signals, the highest accuracy was achieved using the Manhattan distance (88.75%), highlighted in bold in the table. This value is around 3.75% higher than the second best result that was attained by the Euclidean distance metric (85%).

In Table 2 is shown the confusion matrix of the classification means for the 4-MHz frequency backscattered signals; one can observe the difference between the Euclidean and Manhattan distances' performance compared to the other distances, since these two distances properly classified an average of 16–18 samples per class, while the other ones confused a large part of the samples, the majority being classified as belonging to the 200-h class.

In Table 3 is presented the confusion matrix of the classification means for the 4-MHz frequency pulse echo signals; one can conclude that the classification was more distributed across the classes, since 7–14 of the samples were classified correctly with the others distributed across the other classes.

In Table 4 is presented the confusion matrix for the 5-MHz frequency backscattered signals. In this case, the performance of the Euclidean and Manhattan distances was around 25% below the one for the frequency backscattered signals, whereas the Euclidean distance has an accuracy between eight and 15 samples of each class, and the Manhattan distance had an accuracy from 10–14. The other distances classified correctly, on average, less than 10 samples per class.

Table 5 shows the confusion matrix for the 5-MHz frequency pulse echo signals. Here, the Manhattan distance achieved a mean accuracy of 11–16 samples, reaching in the best case 70% accuracy and in the worst cases 58.75%, whereas the Euclidean distance achieved a mean accuracy between 10 and 17, reaching a maximum of 75% and a minimum of 61.25%. Notable was the squared chi-squared distance that in its best result achieved an accuracy rate of 63.75%; the other distances achieved a mean accuracy from 1–15.

In Table 6, the training and testing average times for the 4- and 5-MHz frequency signals are presented in milliseconds. All distances achieved analogous results regarding training and testing times for the backscattered signals, keeping a time of 0.2 ms for training and of 0.1 ms for testing. For the pulse echo signals, average times range from 0.5–1 milliseconds for training and 0.2–0.3 for testing.

#### 3.1.2. Samples Aged at 950 °C

In order to ascertain the best classification performance of the samples aged at 950 °C, we analyzed in detail the classification of each round. As shown in Table 7, which indicates the accuracy rates and harmonic mean for the 4- and 5-MHz frequency signals, the Manhattan distance achieved 60% accuracy for the 4-MHz pulse echo and backscattered signals and 60.63% for the 5-MHz pulse echo signals. However, the Euclidean distance used with the 5-MHz pulse echo signals may be considered more suitable for the classification, since in its best case, it achieved 70% accuracy and in its worst case 53.75%, whereas the results of the Manhattan distance for the 4-MHz backscattered signals ranged from 57.5%–60% and for the 4-MHz pulse echo signals from 48.75%–63.75%. All rounds using the 4-MHz backscattered signals with the Euclidean distance achieved an accuracy rate of 57.5%.

In general, the classifications involving the 5-MHz backscattered signals were very unsatisfactory, with accuracy rates below 40%.

Table 8 shows that for the 4-MHz frequency backscattered signals, all distances at some time could accurately classify more than 10 samples as 0 h and 10 h classes, whereas only the Euclidean and Manhattan distances could do the same with the 100 h and 200 h classes.

In Table 9 is presented the confusion matrix of the classification means of the 4-MHz frequency pulse echo signals. It can be observed that only the Euclidean and Manhattan distances achieved more than 50% of classification for each class, whereas the Euclidean distance achieved a maximum of 63.75% and the Manhattan distance a maximum of 60%.

The data in Table 10 show that for the 5-MHz frequency backscattered signals, a poor performance was achieved in general. The Manhattan distance achieved a maximum of 45% and a minimum of 31.25% accuracy, whereas the Euclidean distance achieved a maximum and a minimum of 37.5% and 36.25%, respectively; the other ones kept below 35%.

Table 11 shows the confusion matrix of the classification means of the 5-MHz frequency pulse echo signals. The data presented show that the best performance for the temperature of 950 °C was achieved by the Euclidean distance with a maximum of 70%, followed by the Manhattan distance with 68.75%; the others remained between 57.5% and 22.5%.

In Table 12 is shown the training and testing average times in milliseconds for the 4- and 5-MHz frequency signals. All distances achieved analogous results for the training and testing times for the backscattered signals, keeping a time of 0.2 ms for training and of 0.1 for testing. For the pulse echo signals, the average times range from 0.2–0.4 ms for training and 0.1 for testing.

#### 3.1.3. Samples Aged at 650 and 950 °C

For this dataset, as can be seen in Table 13, the best accuracy was achieved by the Euclidean distance, with a value of 65.86%, but the highest harmonic mean belongs to the Manhattan metric, with 83.5%. This is due to the fact that in some rounds, the classification performance achieved by the Manhattan distance was higher than the one achieved by the Euclidean distance. The best classification of the Manhattan distance was 71.4%, whereas the best accuracy achieved by the Euclidean distance was equal to 67.86%. The other distances achieved accuracy rates lower than 30%, with the chi-squared distance achieving in its worst classification a value of 10.71%.

Regarding the processing times, indicated in Table 14, the distances that classified the samples correctly more often were those that took longer to do the training, with the Manhattan distance taking between 0.5 and 1 ms and the Euclidean distance from 0.4–0.6 ms. For the test, the Manhattan distance took from 0.16–0.3 ms, whereas the Euclidean distance took from 0.18–0.24 ms. The pulse echo signals were those that took longer to train the classifier, taking around 0.8 ms for all distances.

For the 4-MHz backscattered signals, as shown in Table 15, the Manhattan distance could classify correctly many of the samples related to the signals associated with the temperature of 650 °C, with an average from 15–18.5 samples classified correctly, whereas for the signals associated with the temperature of 950 °C, it achieved an average of correct classifications from 7.5–11 samples. The same applies to the Euclidean distance, that classified correctly from 10–15 samples of the classes associated with the temperature of 650 °C and 7–13 samples for the classes related to the temperature of 950 °C. The remaining distances confused considerably the data associated with the 650 °C/200 h class, with an average of 11–18 samples in all classes classified as belonging to this class.

With the 4-MHz frequency pulse echo signals, Table 16, the classification was more confusing, with wrong classifications for all classes and distances. The Manhattan distance achieved an average accuracy in most of its classes from 8–13.5 samples, whereas the Euclidean distance achieved an average accuracy from 6.5–13 samples. The squared chi-squared and Canberra distances achieved similar results, with an average of samples classified correctly between 5 and 12.5, whereas the Bray–Curtis distance from 5–10.5 and the chi-squared distance below 10 samples.

Table 17 presents the result of the confusion matrix for the 5-MHz frequency backscattered signals, and it can be observed that all distances had bad performances; with the exception of the 650 °C/10 h class, for which the Manhattan and Euclidean distances have the best accuracy rate of 42.14% and 40.71%, respectively. The distance for others had the best results below 30%.

From Table 18, which shows the confusion matrix for the 5-MHz frequency pulse echo signals, it can be confirmed that the performance of all distances was better than the ones presented in Table 17, but still lower than the ones shown in Table 15. The Manhattan distance achieved an average of classification varying from 9–15 correct samples per class, with a maximum accuracy of 64.29% and a minimum accuracy of 48.57%. The Euclidean distance achieved an average of accurate correct classification varying from 9–14.5 samples per class, with a maximum accuracy of 59.29% and a minimum accuracy of 47.14%. The squared chi-squared metric achieved a maximum accuracy of 47.86% and a minimum accuracy of 34.29%. The Canberra distance achieved a maximum accuracy of 36.42% and a minimum accuracy of 29.29%; the Bra–Curtis distance achieved a maximum accuracy of 30% and a minimum accuracy of 22.14%; and finally, the chi-squared distance achieved a maximum accuracy of 22.14% and a minimum accuracy of 11.43%.

## 4. Conclusions

This work evaluated the efficiency and efficacy of the OPF classifier configured with six distance functions to classify ultrasonic signals, raw data background echo and backscattered signals acquired at frequencies of 4 and 5 MHz, to characterize the phase transformations on a Nb-base alloy, thermally aged at 650 and 950 °C for 10, 100 and 200 h, as well as in the as-welded condition.

In regard to this work, the following conclusions can be pointed out:

(1) The results revealed that the classification of the ultrasonic signals using the OPF classifier was sensitive to the microstructural changes occurring in the inconel 625 alloy and that the formation of the secondary phases during the welding process, as well as the phase transformation kinetics due to the different thermal aging times can be efficiently identified;
(2) The best accuracy rates for the thermal aging at 650 °C were obtained using the OPF configured with the Manhattan distance on the backscattered signals acquired with a 4-MHz transducer (accuracy of 88.75%, in 0.384 ms, with a harmonic mean of 61.9);
(3) For the thermal aging at 950 °C, the best results were obtained using the OPF with Euclidean distance on the background echo signals acquired with the 5-MHz transducer (accuracy of 60%, in 0.569 ms, with a harmonic mean of 69.19);
(4) For the thermal aging at 650 and 950 °C, the best results were obtained using the OPF with the Euclidean distance on the backscattered signals acquired with the 4-MHz transducer (accuracy of 67.86%, in 0.713 ms, with a harmonic mean of 80.61);

Based on the results obtained for accurately classifying the ultrasonic signals, it is possible to confirm that the OPF classifier is able to assess the aging conditions to which the inconel 625 alloy is submitted, making it possible to detect the best moment to carry out maintenance services, reducing the costs and maintenance time.

## Figures and Tables

**Table 1:** Accuracy rate and harmonic mean for 4- and 5-MHz frequency signals at 650 °C.

**Frequency(MHz)**	**Signal**		**Manhattan**	**Euclidean**	**Squared Chi-Squared**
4	Backscattered	Acc	88.75 ± 2.11	85 ± 5.43	41.25 ± 4.22
		MH	61.9 ± 1.3	89.52 ± 4.23	44.97 ± 13.29
	Background echo	Acc	60.63 ± 3.54	58.13 ± 6.1	59.38 ± 4.92
		MH	67.89 ± 8.98	68.04 ± 10.66	69.34 ± 7.7

5	Backscattered	Acc	58.75 ± 1.29	60 ± 0	32.5 ± 3.65
		MH	66.31 ± 9.18	69.81 ± 10.27	46.47 ± 8.62
	Background echo	Acc	65.63 ± 4.1	64.38 ± 4.26	56.25 ± 4.72
		MH	74.86 ± 7.39	75.88 ± 9.08	67.19 ± 14.04

**Frequency (MHz)**	**Signal**		**Canberra**	**Bray–Curtis**	**Chi-Square**

4	Backscattered	Acc	30 ± 1.94	27.5 ± 2.84	18.75 ± 3.76
		MH	13.76 ± 4.94	15.08 ± 11.6	9.5 ± 6.16
	Background echo	Acc	53.75 ± 4.77	51.88 ± 3.87	45 ± 5.5
		MH	62.24 ± 9.81	61.87 ± 7.20	58.39 ± 8.03

5	Backscattered	Acc	29.38 ± 3.61	26.25 ± 3.23	31.88 ± 4.21
		MH	43.23 ± 20.08	37.8 ± 2.3	44.42 ± 10.36
	Background echo	Acc	48.13 ± 4.02	40.63 ± 4.49	26.25 ± 5.16
		MH	53.83 ± 16.29	51.01 ± 17.25	35.35 ± 10.92

**Table 2:** Confusion matrix of the classification means for the 4-MHz frequency backscattered signals at 650 °C.

**True Class**	**Classified as**	**Manhattan**	**Euclidean**	**Squared Chi-Squared**	**Canberra**	**Bray Curtis**	**Chi Square**
0h	0h	17	18	5	2	1	1
	10 h	1	1	2	0	1	1
	100 h	2	1	2	1	1	1
	200 h	0	0	11	17	17	17

10 h	0h	1	0	1	1	0	0
	10 h	18	18	2	1	0	1
	100 h	1	2	1	0	0	0
	200 h	0	0	16	18	20	19

100 h	0h	0	1	0.5	0	1	1
	10 h	2	3	1.5	0	0	0
	100 h	18	16	7	1	3	1
	200 h	0	0	11	19	16	18

200 h	0h	1	0	0.5	0	1	1
	10 h	0	1	1.5	0	0	1
	100 h	1	1	1	1	1	4
	200 h	18	18	17	19	18	14

**Table 3:** Confusion matrix of the classification means for the 4-MHz frequency pulse echo signals at 650 °C.

**True Class**	**Classified as**	**Manhattan**	**Euclidean**	**Squared Chi-Squared**	**Canberra**	**Bray Curtis**	**Chi Square**
0h	0h	10	9	11.5	9.5	9	9.5
	10 h	4	6	3.5	4.5	6	4
	100 h	2	1	2	2	1	3
	200 h	2	4	3	4	4	3.5

10 h	0h	3	3.5	3	3.5	3.5	4
	10 h	12	12	12	11.5	12	10
	100 h	2	2	2	2	2	1
	200 h	3	2.5	3	3	2.5	5

100 h	0h	1.5	2.5	1.5	2	2.5	4
	10 h	2.5	4.5	1	1.5	4.5	2.5
	100 h	16	12	16	14.5	12	11.5
	200 h	0	1	1.5	2	1	2

200 h	0h	3.5	5	4.5	5	5	5
	10 h	2.5	5	3	4	5	6.5
	100 h	3	2	3	1	2	1
	200 h	11	8	9.5	10	8	7.5

**Table 4:** Confusion matrix of the classification means for the 5-MHz frequency backscattered signals at 650 °C.

**True Class**	**Classified as**	**Manhattan**	**Euclidean**	**Squared Chi-Squared**	**Canberra**	**Bray Curtis**	**Chi Square**
0h	0h	11	14	7	9	5	9
	10 h	9	6	6	6.5	6	2
	100 h	0	0	7	3	6	5
	200 h	0	0	0	1.5	3	4

10 h	0h	2	0	8	7	5	5
	10 h	12	11	4	1	5	5
	100 h	6	9	6	5	5	3
	200 h	0	0	2	7	5	7

100 h	0h	1	2	4	7	4	5
	10 h	9	3	5	6	5	3
	100 h	10	15	8	5	6	5
	200 h	0	0	3	2	5	7

200 h	0h	1	2	3	1	5	4
	10 h	3	4	6	4	4	4
	100 h	2	6	5	6	6	4
	200 h	14	8	6	9	5	8

**Table 5:** Confusion matrix of the classification means for the 5-MHz frequency pulse echo signals at 650 °C.

**True Class**	**Classified as**	**Manhattan**	**Euclidean**	**Squared Chi-Squared**	**Canberra**	**Bray Curtis**	**Chi Square**
0h	0h	14	14	15.5	9	9	4
	10 h	3	4	3.5	3	2	6
	100 h	1	1	0	3	4	4
	200 h	2	1	1	5	5.5	6

10 h	0h	4	3	3	2.5	2	4
	10 h	11	13	8	6.5	4	4
	100 h	3	3	4	5	6	5
	200 h	2	1	5	6	8	7

100 h	0h	0.5	1	3	1	2	3
	10 h	2	1	1	2	2	4
	100 h	16	17	16	16	13	7
	200 h	1.5	1	1	1	3	6

200 h	0h	2	3	3.5	3.5	4.5	5
	10 h	2	3.5	4	5.5	5.5	4
	100 h	3	3	3	2	4	5
	200 h	13	10.5	9.5	9	6	6

**Table 6:** Average of training and testing times in milliseconds for the 4- and 5-MHz frequency signals at 650 °C.

**Frequency (MHz)**	**Signal**	**Time**	**Manhattan**	**Euclidean**	**Squared Chi-Squared**
4	Backscattered	Train	0.279 ± 0.159	0.355 ± 3.912	0.236 ± 0.092
		Test	0.105 ± 0.056	0.097 ± 0.031	0.090 ± 0.063
	Background echo	Train	0.512 ± 0.015	0.312 ± 0.063	1.050 ± 2.680
		Test	0.162 ± 0.035	0.126 ± 0.040	0.226 ± 0.293

5	Backscattered	Train	0.3 ± 0.179	0.288 ± 0.174	0.258 ± 0.131
		Test	0.095 ± 0.074	0.125 ± 0.049	0.143 ± 0.041
	Background echo	Acc	0.520 ± 0.097	0.446 ± 9.254	0.376 ± 0.283
		Test	0.285 ± 0.041	0.192 ± 0.054	0.191 ± 0.056

**Frequency (MHz)**	**Signal**	**Time**	**Canberra**	**Bray–Curtis**	**Chi-Square**

4	Backscattered	Train	0.205 ± 0.016	0.209 ± 0.030	0.216 ± 0.136
		Test	0.095 ± 0.018	0.113 ± 0.013	0.090 ± 0.021
	Background echo	Acc	0.593 ± 0.392	0.707 ± 0.359	0.398 ± 0.066
		Test	0.185 ± 0.047	0.247 ± 0.141	0.152 ± 0.035

5	Backscattered	Train	0.229 ± 0.010	0.329 ± 0.129	0.238 ± 0.116
		Test	0.091 ± 0.009	0.158 ± 0.080	0.113 ± 0.036
	Background echo	Acc	0.542 ± 0.449	0.388 ± 0.047	0.525 ± 0.166
		Test	0.324 ± 0.102	0.166 ± 0.036	0.237 ± 0.050

**Table 7:** Accuracy rates and harmonic mean for the 4- and 5-MHz frequency at 950 °C.

**Frequency (MHz)**	**Signal**		**Manhattan**	**Euclidean**	**Squared Chi-Squared**
4	Backscattered	Acc	60 ± 1.05	57.5 ± 0	38.75 ± 1.44
		MH	71.36 ± 7.28	70.8 ± 16.65	50.86 ± 3.74
	Background echo	Acc	60 ± 4.75	56.88 ± 4.19	55 ± 4.02
		MH	69.92 ± 4.97	64.65 ± 6.2	62.03 ± 10.31

5	Backscattered	Acc	38.75 ± 4	36.88 ± 0.66	25 ± 2.77
		MH	53.95 ± 7.55	11.67 ± 22.99	34.64 ± 8.21
	Background echo	Acc	60.63 ± 5.24	60 ± 4.87	48.75 ± 4.42
		MH	69.94 ± 6.61	69.19 ± 8.68	59.75 ± 10.53

**Frequency (MHz)**	**Signal**		**Canberra**	**Bray–Curtis**	**Chi-Square**

4	Backscattered	Acc	30 ± 2.64	25 ± 0.53	34.38 ± 3.7
		MH	43.32 ± 4.38	33.78 ± 13.03	48.77 ± 6.74
	Background echo	Acc	50.63 ± 2.83	46.25 ± 3.59	43.13 ± 6.82
		MH	59.98 ± 8.54	56.9 ± 10.87	55.12 ± 7.58

5	Backscattered	Acc	23.75 ± 2.49	31.25 ± 2.13	32.5 ± 4.49
		MH	34.28 ± 11.93	47.36 ± 7.76	47.65 ± 8.96
	Background echo	Acc	39.38 ± 4.19	34.38 ± 3.76	28.13 ± 4.05
		MH	49.66 ± 13.32	44 ± 5.4	41.63 ± 2.66

**Table 8:** Confusion matrix of the classification means for the 4-MHz frequency backscattered signals at 950 °C.

**True Class**	**Classified as**	**Manhattan**	**Euclidean**	**Squared Chi-Squared**	**Canberra**	**Bray Curtis**	**Chi Square**
0h	0h	14	15	9	5	6	5
	10 h	3	1	3	2	6	3
	100 h	2	2	5	10	3	6
	200 h	1	2	3	3	5	6

10 h	0h	0	1	2	3	7	2
	10 h	12	6	8	6	4	8
	100 h	7	10	6	8	5	5
	200 h	1	2	3	3	4	5

100 h	0h	2	1	6	5.5	6	1
	10 h	7	5	5	4.5	3	4
	100 h	10	13	7	6	3	9
	200 h	1	1	2	4	8	6

200 h	0h	4	1.5	4	2.5	3	2
	10 h	1	2.5	5	4	5	4.5
	100 h	3	4	3	7	4	6
	200 h	12	12	8	6.5	8	7.5

**Table 9:** Confusion matrix of the classification means for the 4-MHz frequency pulse echo signals at 950 °C.

**True Class**	**Classified as**	**Manhattan**	**Euclidean**	**Squared Chi-Squared**	**Canberra**	**Bray Curtis**	**Chi Square**
0h	0h	12	10.5	10	9.5	9.5	10
	10 h	2	2	2.5	5	4	4.5
	100 h	4	4	3	2	3	4.5
	200 h	2	3.5	4.5	3.5	3.5	1

10 h	0h	2	1	1	2	2	3.5
	10 h	14.5	13.5	15	15	14	11.5
	100 h	2	3	2	2	2	4
	200 h	1.5	2.5	2	1	2	1

100 h	0h	4.5	3.5	5	5.5	6	6
	10 h	3	3.5	5	5.5	6	5
	100 h	12	11	8	8	7	7
	200 h	0.5	2	2	1	1	2

200 h	0h	3.5	4.5	4.5	4.5	5	4
	10 h	3	2	3	3	3	4
	100 h	2	3	2	2	3	4
	200 h	11.5	10.5	10.5	11.5	8	8

**Table 10:** Confusion matrix of the classification means for the 5-MHz frequency backscattered signals at 950 °C.

**True Class**	**Classified as**	**Manhattan**	**Euclidean**	**Squared Chi-Squared**	**Canberra**	**Bray Curtis**	**Chi Square**
0h	0h	6	13	4	7	7	7
	10 h	1	2	7.5	3.5	5	6
	100 h	2	2	5	4.5	4	4
	200 h	11	3	3.5	5	4	3

10 h	0h	1	2.5	3	6	5	3
	10 h	8	6.5	7	6	7	8
	100 h	4	6	6	5	4	5
	200 h	7	5	4	3	4	4

100 h	0h	3	3.5	5	5	5	4
	10 h	3	5.5	6	7	6	5
	100 h	8	5	5	3	7	9
	200 h	6	6	4	5	2	2

200 h	0h	2	3	4	5	4	3
	10 h	6	9.5	7	3	7	7
	100 h	2	2	6	9	5	6
	200 h	10	5.5	3	3	4	4

**Table 11:** Confusion matrix of the classification means for the 5-MHz frequency pulse echo signals at 950 °C.

**True Class**	**Classified as**	**Manhattan**	**Euclidean**	**Squared Chi-Squared**	**Canberra**	**Bray Curtis**	**Chi Square**
0h	0h	15	17	14	12	9	5.5
	10 h	2	1	1	1	1	3.5
	100 h	2	1	3	4	5	5
	200 h	1	1	2	3	5	6

10 h	0h	1.5	1	1	3.5	3	5
	10 h	13.5	12.5	8	5.5	6	6
	100 h	4	4	8	7	7	6
	200 h	1	2.5	3	4	4	3

100 h	0h	3	4	3	5.5	7	5
	10 h	5	5	5	4.5	4	7
	100 h	10	10	10	7	5	5
	200 h	2	1	2	3	4	3

200 h	0h	3	4	2.5	5	5.5	6
	10 h	4	3	6	4	4.5	2.5
	100 h	2	1	2	3	3	5
	200 h	11	12	9.5	8	7	6.5

**Table 12:** Average of training and testing times in milliseconds for the 4- and 5-MHz frequency signals at 950 °C.

**Frequency (MHz)**	**Signal**	**Time**	**Manhattan**	**Euclidean**	**Squared Chi-Squared**
4	Backscattered	Train	0.254 ± 0.123	0.232 ± 0.007	0.233 ± 0.152
		Test	0.098 ± 0.040	0.084 ± 0.016	0.091 ± 0.035
	Background echo	Train	0.282 ± 0.055	0.252 ± 0.029	0.629 ± 0.394
		Test	0.092 ± 0.054	0.092 ± 0.003	0.221 ± 0.075

5	Backscattered	Train	0.271 ± 0.097	0.240 ± 0.107	0.284 ± 0.122
		Test	0.090 ± 0.032	0.106 ± 0.033	0.118 ± 0.060
	Background echo	Acc	0.397 ± 0.086	0.415 ± 0.132	0.393 ± 0.240
		Test	0.195 ± 0.090	0.154 ± 0.040	0.189 ± 0.030

**Frequency (MHz)**	**Signal**	**Time**	**Canberra**	**Bray–Curtis**	**Chi-Square**

4	Backscattered	Train	0.234 ± 0.188	0.322 ± 0.151	0.219 ± 0.087
		Test	0.095 ± 0.037	0.100 ± 0.032	0.092 ± 0.024
	Background echo	Acc	0.262 ± 0.047	0.478 ± 0.146	0.255 ± 0.024
		Test	0.100 ± 0.114	0.242 ± 0.100	0.095 ± 0.013

5	Backscattered	Train	0.231 ± 0.105	0.339 ± 0.120	0.224 ± 0.133
		Test	0.096 ± 0.055	0.113 ± 0.044	0.082 ± 0.078
	Background echo	Acc	0.379 ± 0.061	0.399 ± 0.070	0.369 ± 0.070
		Test	0.201 ± 0.058	0.186 ± 0.038	0.182 ± 0.072

**Table 13:** Accuracy rates and harmonic mean for the 4- and 5-MHz frequency signals at 650 and 950 °C.

**Frequency (MHz)**	**Signal**		**Manhattan**	**Euclidean**	**Squared Chi-Squared**
4	Backscattered	Acc	65.71 ± 2.94	67.86 ± 3.32	27.14 ± 1.74
		MH	83.50 ± 16.26	80.61 ± 14.81	25.91 ± 14.31
	Background echo	Acc	46.43 ± 3.33	46.43 ± 3.28	46.43 ± 3.9
		MH	56.47 ± 7.59	60.09 ± 8.65	55.77 ± 8.92

5	Backscattered	Acc	30.71 ± 5.9	39.29 ± 1.51	19.29 ± 2.82
		MH	46.15 ± 10.03	50.33 ± 7.25	25.34 ± 13.79
	Background echo	Acc	55 ± 4.31	53.21 ± 3.54	41.07 ± 3.52
		MH	66.76 ± 8.48	67.2 ± 7.97	50.33 ± 13.58

**Frequency (MHz)**	**Signal**		**Canberra**	**Bray–Curtis**	**Chi-Square**

4	Backscattered	Acc	18.57 ± 1.32	16.43 ± 1.59	13.21 ± 1.73
		MH	13.93 ± 9.69	9.5 ± 13.16	9.51 ± 11.67
	Background echo	Acc	39.29 ± 3.35	36.07 ± 2.79	28.93 ± 3.46
		MH	50.78 ± 12.36	46.65 ± 9.67	45.26 ± 10.59

5	Backscattered	Acc	17.14 ± 1.84	26.43 ± 1.38	21.07 ± 2.18
		MH	25.18 ± 9.42	39.44 ± 10.73	35.36 ± 13.35
	Background echo	Acc	35 ± 2.46	27.5 ± 2.69	16.43 ± 3.17
		MH	39.29 ± 16.7	36.16 ± 14.77	25.76 ± 6.68

**Table 14:** Average of training and testing times in milliseconds for the 4- and 5-MHz frequency signals at 650 and 950 °C.

**Frequency (MHz)**	**Signal**	**Time**	**Manhattan**	**Euclidean**	**Squared Chi-Squared**
4	Backscattered	Train	0.512 ± 0.015	0.528 ± 0.204	0.411 ± 0.014
		Test	0.162 ± 0.035	0.185 ± 0.048	0.192 ± 0.075
	Background echo	Train	0.945 ± 0.211	0.599 ± 0.099	0.888 ± 6.323
		Test	0.333 ± 0.108	0.243 ± 0.056	0.290 ± 0.080

5	Backscattered	Train	0.555 ± 0.182	0.454 ± 0.218	0.456 ± 0.197
		Test	0.192 ± 0.050	0.188 ± 0.027	0.219 ± 0.063
	Background echo	Acc	0.638 ± 0.075	0.492 ± 0.017	0.751 ± 0.136
		Test	0.265 ± 0.076	0.182 ± 0.070	0.360 ± 0.205

**Frequency (MHz)**	**Signal**	**Time**	**Canberra**	**Bray–Curtis**	**Chi-Square**

4	Backscattered	Train	0.386 ± 0.019	0.387 ± 0.047	0.387 ± 0.022
		Test	0.192 ± 0.015	0.189 ± 0.016	0.197 ± 0.035
	Background echo	Acc	0.957 ± 0.285	0.804 ± 0.222	0.899 ± 7.768
		Test	0.371 ± 0.105	0.411 ± 0.100	0.303 ± 0.160

5	Backscattered	Train	0.453 ± 0.126	0.410 ± 0.032	0.410 ± 0.080
		Test	0.205 ± 0.022	0.198 ± 0.040	0.205 ± 0.026
	Background echo	Acc	0.581 ± 0.054	0.800 ± 0.445	0.437 ± 0.040
		Test	0.264 ± 0.044	0.380 ± 0.189	0.195 ± 0.036

**Table 15:** Confusion matrix of the classification means for the 4-MHz frequency backscattered signals at 650 and 950 °C.

**True Class**	**Classified as**	**Manhattan**	**Euclidean**	**Squared Chi-Squared**	**Canberra**	**Bray Curtis**	**Chi Square**
0h	0h	15	18	3.5	2.5	1	1
	650 °C/10 h	1	1	2	1	1	0
	650 °C/100 h	0.5	1	1	1	1	1
	650 °C/200 h	0	0	11	13	16	13
	950 °C/10 h	2	0	1	0	0	1
	950 °C/100 h	0	0	1	1	0	2.5
	950 °C/200 h	2	0	1	0.5	0	1

650 °C/10 h	0h	0	0	0	0	0	0.5
	650 °C/10 h	15	14	3	1	0	1
	650 °C/100 h	1	3	2	1	0	0
	650 °C/200 h	0	0	13	16	17	15
	950 °C/10 h	1.5	1	0	1	0	1
	950 °C/100 h	1	2	1.5	0	2	1
	950 °C/200 h	0.5	0	0	1	0	2

650 °C/100 h	0h	0	0	0	0	0	0.5
	650 °C/10 h	3	2	1	0	0	1
	650 °C/100 h	16.5	18	7	2	4	1
	650 °C/200 h	0	0	11.5	16	15	14
	950 °C/10 h	0	0	0	0	0	1
	950 °C/100 h	0	0	0	0	0	2.5
	950 °C/200 h	0.5	0	0	0	0	2

650 °C/200 h	0h	0	0	0	1	0	1
	650 °C/10 h	1	1	1	0	1	1.5
	650 °C/100 h	0.5	1	1	0	2	2
	650 °C/200 h	18.5	16	16	17	16	8.5
	950 °C/10 h	0	0	0	0	0	2
	950 °C/100 h	0	0	1	1	0	2.5
	950 °C/200 h	0	2	0	1	0	3.5

950 °C/10 h	0h	2	2	0	0	1	1
	650 °C/10 h	1	0	0	0	0	1
	650 °C/100 h	0	0	0	0	1	0
	650 °C/200 h	0	0	12	15	18	11.5
	950 °C/10 h	7.5	8	2.5	2	0	3.5
	950 °C/100 h	8	9	2	1	1	2
	950 °C/200 h	2	1	1	0.5	0	1.5

950 °C/100 h	0h	0	2	2	1	0	0
	650 °C/10 h	0.5	1	1	0	0	0.5
	650 °C/100 h	0	0	0	0	0	1
	650 °C/200 h	0	0	12	17	18	13
	950 °C/10 h	7	6	2	0	0.5	1
	950 °C/100 h	11	10	2	1.5	1	3
	950 °C/200 h	2	1	1	0	0	1

950 °C/200 h	0h	3	2	2	2	0	1
	650 °C/10 h	1	1	0	0	0	2.5
	650 °C/100 h	0.5	0	0	0	1	1
	650 °C/200 h	0	0	13	16	17	15
	950 °C/10 h	4	3	1	1	1	0.5
	950 °C/100 h	2.5	3	1	1	0.5	1
	950 °C/200 h	9.5	11	3	0	0.5	1

**Table 16:** Confusion matrix of the classification means for the 4-MHz frequency pulse echo signals at 650 and 950 °C.

**True Class**	**Classified as**	**Manhattan**	**Euclidean**	**Squared Chi-Squared**	**Canberra**	**Bray Curtis**	**Chi Square**
0h	0h	9.5	10.5	7.5	6	5	7
	650 °C/10 h	2	2.5	3.5	2.5	3	2.5
	650 °C/100 h	1.5	2	1	2.5	2	2.5
	650 °C/200 h	1.5	2	2	3	3	3
	950 °C/10 h	2	1	1.5	3.5	4	1.5
	950 °C/100 h	1	1	3.5	1.5	2	2.5
	950 °C/200 h	2.5	1	1	2	2	1

650 °C/10 h	0h	2	2	2.5	3	2	2
	650 °C/10 h	9	7.5	10	9	8	4
	650 °C/100 h	2	1.5	2.5	2.5	1.5	1
	650 °C/200 h	1	2	1	1.5	2	5
	950 °C/10 h	3	4	3.5	2	3.5	3
	950 °C/100 h	2	2	0	1	1.5	2
	950 °C/200 h	1	1	1.5	1	1.5	2

650 °C/100 h	0h	1	1.5	2	1	1	1
	650 °C/10 h	1	1	1	2	2	2.5
	650 °C/100 h	13.5	13	11	12.5	9	9
	650 °C/200 h	1	0	1	1	2	2
	950 °C/10 h	1.5	2	2	1	3.5	2
	950 °C/100 h	1	1	1.5	1.5	1	2
	950 °C/200 h	1	1	1.5	1	1	1.5

650 °C/200 h	0h	2	2	1.5	3	4	3
	650 °C/10 h	3	2	2	2.5	2	3
	650 °C/100 h	1	1	1	2	1	2
	650 °C/200 h	8.5	9	8.5	5.5	5	5
	950 °C/10 h	2	2	3	3	3	3.5
	950 °C/100 h	2	2	2	2	3	1
	950 °C/200 h	1.5	2	2.5	2	1	2.5

950 °C/10 h	0h	1	1	1	1	1.5	1.5
	650 °C/10 h	2	1.5	1	1.5	2	3
	650 °C/100 h	2.5	1.5	1.5	1.5	1	3
	650 °C/200 h	1.5	2	1	1.5	2.5	2.5
	950 °C/10 h	10.5	10.5	12.5	11	11.5	8
	950 °C/100 h	1.5	2.5	2	1.5	1	2
	950 °C/200 h	1	1	1	1	0.5	0

950 °C/100 h	0h	3.5	2	4.5	3	3.5	4
	650 °C/10 h	2	2	1.5	2.5	2.5	3
	650 °C/100 h	1	2	1.5	1.5	2	1.5
	650 °C/200 h	1.5	1.5	1.5	1.5	2	1.5
	950 °C/10 h	3	3.5	4	3	4	4
	950 °C/100 h	8.5	8.5	7	7.5	5.5	4
	950 °C/200 h	0.5	0.5	0	1	1	2

950 °C/200 h	0h	2.5	3	2.5	3	3.5	3.5
	650 °C/10 h	2	1.5	1.5	2.5	3.5	2.5
	650 °C/100 h	2	2	2	1	1.5	1
	650 °C/200 h	2	2	2	2	2	3
	950 °C/10 h	1.5	1.5	2	2.5	3	2
	950 °C/100 h	1.5	1.5	2	2	0.5	1
	950 °C/200 h	8.5	8.5	8	7	6	7

**Table 17:** Confusion matrix of the classification means for the 5-MHz frequency backscattered signals at 650 and 950 °C.

**True Class**	**Classified as**	**Manhattan**	**Euclidean**	**Squared Chi-Squared**	**Canberra**	**Bray Curtis**	**Chi Square**
0h	0h	6	7	3.5	3	5	6
	650 °C/10 h	4	3.5	2.5	2	0	2
	650 °C/100 h	2	1	3	1	2	2
	650 °C/200 h	0	0	1	0	0	3
	950 °C/10 h	2	1.5	4	5	4	3
	950 °C/100 h	1	2	3	5	5	3
	950 °C/200 h	5	5	3	4	2	1

650 °C/10 h	0h	3	5	3	4	1	2
	650 °C/10 h	11.5	10	2	3	5	2
	650 °C/100 h	4.5	4	4.5	0.5	2	2.5
	650 °C/200 h	0	0	1	4	4	4
	950 °C/10 h	0	0	4	3	2	4
	950 °C/100 h	0	0.5	3	2.5	3	3.5
	950 °C/200 h	1	0.5	2.5	3	3	3

650 °C/100 h	0h	3	2	3	2.5	1.5	2
	650 °C/10 h	9	9	2	3	1.5	1
	650 °C/100 h	8	8.5	3.5	2.5	3	3
	650 °C/200 h	0	0	0	1	3	4
	950 °C/10 h	0	0	5	6	4	4
	950 °C/100 h	0	0	3	2	4	3
	950 °C/200 h	0	0.5	3.5	3	3	3

650 °C/200 h	0h	1.5	0.5	1	1.5	2.5	3
	650 °C/10 h	5	5	5	5	2	3
	650 °C/100 h	5	3.5	3	3	1	3
	650 °C/200 h	8	9.5	5	4	8.5	6.5
	950 °C/10 h	0.5	0	2	3	1	1
	950 °C/100 h	0	0.5	2	2.5	2	2
	950 °C/200 h	0	1	2	1	3	1.5

950 °C/10 h	0h	1	1	4	2.5	3	2
	650 °C/10 h	0.5	0	1.5	1.5	0	1
	650 °C/100 h	0.5	0	2.5	2	3	2
	650 °C/200 h	0	0	0	1	1.5	2.5
	950 °C/10 h	6	6.5	6	6	5	5.5
	950 °C/100 h	6	5.5	3	4	3.5	5
	950 °C/200 h	6	7	3	3	4	2

950 °C/100 h	0h	2	3.5	5	3	3.5	4
	650 °C/10 h	0	1	1.5	1	5	1
	650 °C/100 h	0	0	2	2	2	1
	650 °C/200 h	0	0	0	0	0.5	1
	950 °C/10 h	6	4.5	3	4.5	2	3.5
	950 °C/100 h	8	7	3.5	3.5	6	6
	950 °C/200 h	4	4	5	5	1	2.5

950 °C/200 h	0h	3.5	4	4.5	3	5	3
	650 °C/10 h	0.5	1	2.5	1	1	1.5
	650 °C/100 h	1	0.5	2	1	0.5	1.5
	650 °C/200 h	0	0	0	0	2	4
	950 °C/10 h	5	4	6	6	5	4.5
	950 °C/100 h	3	4	4	7	3.5	3
	950 °C/200 h	7	6.5	1	2	3	2.5

**Table 18:** Confusion matrix of the classification means for the 5-MHz frequency pulse echo signals at 650 and 950 °C.

**True Class**	**Classified as**	**Manhattan**	**Euclidean**	**Squared Chi-Squared**	**Canberra**	**Bray Curtis**	**Chi Square**
0h	0h	14	13	12.5	8	5	3.5
	650 °C/10 h	2	3	1.5	3	2	3.5
	650 °C/100 h	1	1	1.5	2.5	2	3
	650 °C/200 h	1	1.5	0	3.5	4	4
	950 °C/10 h	1	1	1	0	2.5	1
	950 °C/100 h	0.5	0.5	2	3	2	2
	950 °C/200 h	0.5	0	1.5	1.5	2.5	3

650 °C/10 h	0h	3	3	3	1.5	1.5	3
	650 °C/10 h	10.5	10	6	5	4	2.5
	650 °C/100 h	2.5	2	3.5	5	5.5	4
	650 °C/200 h	1	0	2.5	5.5	5.5	5
	950 °C/10 h	1	1	1.5	0	0	0.5
	950 °C/100 h	1	2.5	2	2	2	2
	950 °C/200 h	1	1.5	1.5	1	1.5	3

650 °C/100 h	0h	0.5	1	1.5	1	1.5	1.5
	650 °C/10 h	1.5	1	1	1	1.5	3
	650 °C/100 h	15	14.5	15	14.5	12	6
	650 °C/200 h	1.5	1	1	2	2	5
	950 °C/10 h	0.5	0.5	0	0	1	1
	950 °C/100 h	1	2	1.5	0	0	2
	950 °C/200 h	0	0	0.5	1.5	2	1.5

650 °C/200 h	0h	1.5	2	3	2	3	4
	650 °C/10 h	2	2.5	2.5	4	3	3
	650 °C/100 h	2.5	2.5	1.5	2	2.5	2.5
	650 °C/200 h	9.5	9	6.5	8	5	4.5
	950 °C/10 h	2	1	2	1	2	1
	950 °C/100 h	2	3	3	1.5	3	2
	950 °C/200 h	0.5	0	1.5	1.5	1.5	3

950 °C/10 h	0h	0	1	0.5	1	1	2
	650 °C/10 h	2	2	0.5	1	1	1.5
	650 °C/100 h	1	1	1.5	3	4	3
	650 °C/200 h	1	0.5	2	4.5	3.5	4
	950 °C/10 h	11	10.5	7	4.5	5.5	4
	950 °C/100 h	3	3.5	5.5	4	4	2.5
	950°C/200 h	2	1.5	3	2	1	3

950 °C/100 h	0h	2	2.5	2	2	2	3
	650 °C/10 h	2	2	4	2	3	2
	650 °C/100 h	2	2	2.5	5	4	3
	650 °C/200 h	1	1	1.5	3.5	5	5
	950 °C/10 h	3	2.5	3.5	3	1.5	3
	950 °C/100 h	9	9	5.5	3	3.5	3
	950 °C/200 h	1	1	1	1.5	1	1

950 °C/200 h	0h	2	2	2.5	1.5	2.5	2
	650 °C/10 h	2	1	1.5	2.5	3	3
	650 °C/100 h	0.5	2	2	4.5	4.5	2.5
	650 °C/200 h	1	1	1	5	4.5	7
	950 °C/10 h	3	2.5	2.5	0.5	2.5	2.5
	950 °C/100 h	1.5	1.5	1	1	2	2
	950 °C/200 h	10	11	7.5	5	2.5	3
